# Dental erosion caused by gastroesophageal reflux disease: a case report

**DOI:** 10.4076/1757-1626-2-8018

**Published:** 2009-07-22

**Authors:** Seda Cengiz, M İnanç Cengiz, Y Şinasi Saraç

**Affiliations:** 1 Prosthetic Dentistry, Private PracticeSamsunTurkey; 2 Periodontology, Vezirköprü Oral Health CenterSamsunTurkey; 3 Department of Prosthodontics, Faculty of Dentistry, Ondokuz Mayis UniversitySamsunTurkey

## Abstract

**Introduction:**

Chronic regurgitation of gastric acids in patients with gastroesophageal reflux disease may cause dental erosion, which can lead in combination with attrition or bruxism to extensive loss of coronal tooth tissue.

**Case presentation:**

This clinical report describes treatment of severe tooth wear of a gastroesophageal reflux disease patient who is 54-year-old Turkish male patient. After his medical treatment, severe tooth wear, bruxism and decreased vertical dimensions were determined. The vertical dimension was re-established and maxillary and mandibular anterior and posterior teeth were prepared for metal-ceramic restorations. Metal-ceramic fixed partial dentures were fabricated as full mouth restorations for both maxillary and mandibular arches because of splinting all teeth. And then maxillary stabilization splint was fabricated for his bruxism history.

**Conclusion:**

Significant loss of coronal tooth structure must taken into consideration. Gastroesophageal reflux disease by itself or in combination with attrition, abrasion or bruxism may be responsible for the loss. An extensive diagnostic evaluation is essential for the medical and dental effects of the problem.

## Introduction

Gastroesophageal reflux disease (GERD), is defined as involuntary muscle relaxing of the upper esophageal sphincter, which allows refluxed acid to move upward through the esophagus into the oral cavity [1]. Dental erosion has been reported with varying prevalences in the population and may be as high as 42% [2]. In adults, GERD is a highly prevalent disease with rates ranging from 21% to 56% in different countries [3]. It is difficult to compare prevalence studies because of the different indices used in the various studies and also because of the different teeth assessed in the sample [4].

It was reported that GERD was diagnosed by endoscopy, where visual identification of mucosal inflammation and oseophagitis was used to identify the existence of GERD [5]. The degree of erosion will depend upon how long the disease has been present and the frequency and quantity of regurgitation. Other causes of erosion such as dietary acids should also be considered [6]. Several factors are known to contribute to enamel erosion. It occurs at a pH of approximately 5.5, which is on the acidic side of the neutral point, and may vary depending on the concentrations of calcium and phosphate ions with the saliva [7]. If wear from other factors such as attrition is occurring at the same time as the erosion the tooth wear will be increased [8]. Combinations of erosion, attrition and abrasion are synergistic [9] and may be responsible for the lost tooth structure. The clinical appearance of the various sub-types of tooth wear can vary. Attrition in its purest form is typified by flattened occlusal surfaces which almost appear as if someone has filled the teeth with sandpaper. The presence of hypertrophic masseter muscles is another warning sign of the impact of bruxism. Erosion from dietary or gastric acids forms smooth lesions which typically appear as cupped occlusal/incisal and concave buccal/facial surfaces. When erosion is the dominant factor, the buccal and lingual surfaces of the upper incisors appear smooth and shiny with a generalised loss of anatomy. The tooth surface continually changes as the acid partially dissolves the outer layer of enamel or dentine which then becomes more susceptible to abrasion or attrition [10].

It is recognized that refluxed acid attacks the palatal surfaces of the upper incisor teeth first, later, if the condition continues, erosion of the occlusal surfaces of the posterior teeth in both arches and the labial or buccal surfaces results from an extended period of acid reflux [11,12]. It is suggested that the force of the regurgitation passing from the pharynx into the mouth propels the gastric fluid forward and causes damage first to the palatal surfaces of the maxillary teeth. In addition, the palatal surfaces are also relatively remote from the major salivary glands and the tongue may also be involved by maintaining contact of the gastric fluid against the palatal surfaces of the teeth. The lower teeth are not affected in the early stages as the tongue provides some protection [13]. In more severe cases however, the protection from the tongue is overwhelmed and the pattern of erosion may be more widespread, usually with the occlusal and buccal surfaces of the lower teeth being eroded next [11].

A simplified wear index has been proposed to define wear on four levels; no wear, enamel exposure, mild and severe dentine exposure. The classification is not only dependent upon the severity of the wear but also the age of the patient [14].

The authors concluded that patients diagnosed with GERD were at a high risk of developing dental erosion. The buffering capacity of the stimulated saliva from the control subjects was significantly greater than patients with symptoms of GERD [15]. Also, subjects diagnosed with GERD had significantly higher tooth wear index scores compared with control subjects [2,15]. A number of case reports have identified gastroesophageal reflux as the likely cause of tooth wear [3,16-18]. In contradictory, it was shown that there was no correlation between GERD and clinical parameters [19-20] but, morphometric analysis of the palatal epithelium in GERD patients showed a statistically significant difference from the healthy control group in the study [19]. The results of morphometric analysis showed a significant difference between groups with respect to luminal and basal surface ratio, thickness of epithelium, and amount of fibroblasts/mm^3^. The smaller the value of the relationship between the basal surface and the external surface, the more severe the aggression received by the epithelium. The values obtaied represent an irritation of the palatal epithelium, which in turn is reflected by a deepening of the epithelial crests in the connective tissue and of the connective papillae in the epithelium. Individuals with the more severe cases of reflux have a more compromised epithelium [19].

Oral symptoms associated with GERD are burning mouth syndrome, dental sensitivity, loss of vertical dimension, and esthetic problems [18]. Tooth wear is a multifactorial process. The impact of wear is usually progressive but can be slow. The wear results in shortened clinical crowns and in conjunction with alveolar compensation complicates treatment [10].

This case report presents the clinical findings, diagnosis, medical and dental rehabilitation of a patient with GERD with severe tooth erosion.

## Case presentation

**Figure 1.**
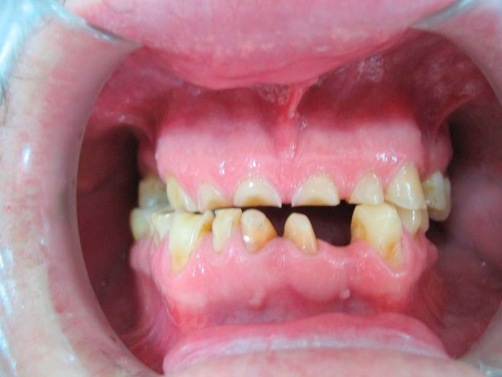
Pre-op intraoral front view in occlusion.

**Figure 2.**
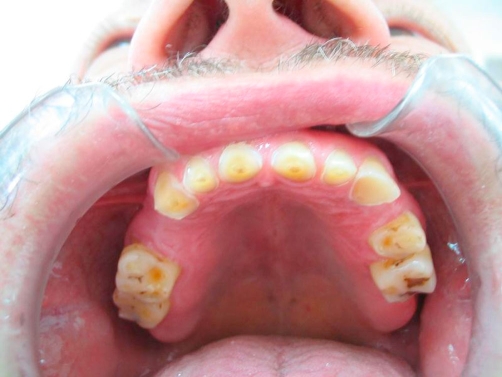
Pre-op intraoral occlusal view of upper arch.

**Figure 3.**
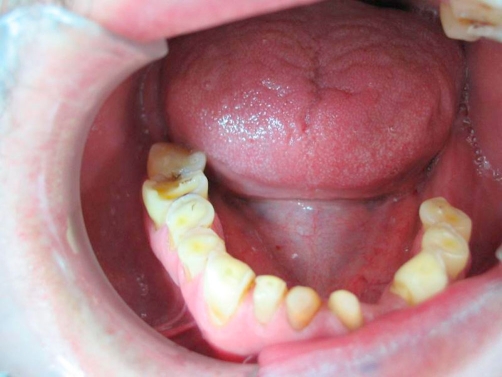
Pre-op intraoral occlusal view of lower arch.

A 54-year-old Turkish man came to our clinic Department of Prosthetic Dentistry, 19 Mayis University, Faculty of Dentistry with the lack of esthetics and function of his teeth. In his medical examination he had gastroesophageal reflux disease history for about 4-5 years and had paid no attention because of any symptoms affecting his life. Also, brushing teeth behaviour was once a day and bruxism history was present. In his clinical examination there was generalize dental erosion including dentin on the occlusal, buccal and palatinal surfaces of maxillary teeth; occlusal and buccal surfaces of mandibular teeth and cervical lesions were seen (Figures 1, 2 and 3) (Smith and Knight Wear Index, Score 3-4) [14]. The questionnaire detailed the patient’s possible exposure to etiologic factors associated with dental erosion, including ingestion of citrus fruits, vinegar and carbonated drinks. The patient did not admit to ingest high consumption of acidic foods and drinks.

**Figure 4.**
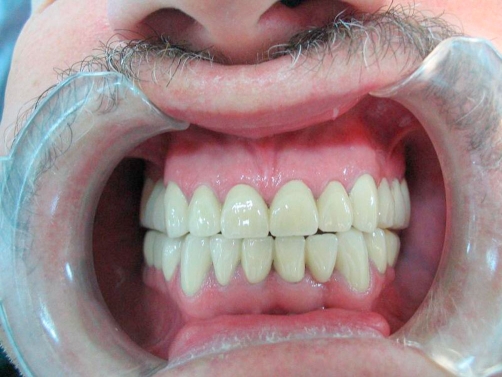
Post-op intraoral front view in occlusion.

**Figure 5.**
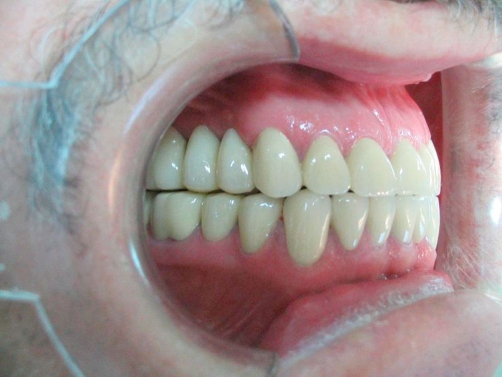
Post-op intraoral right lateral view in occlusion.

**Figure 6.**
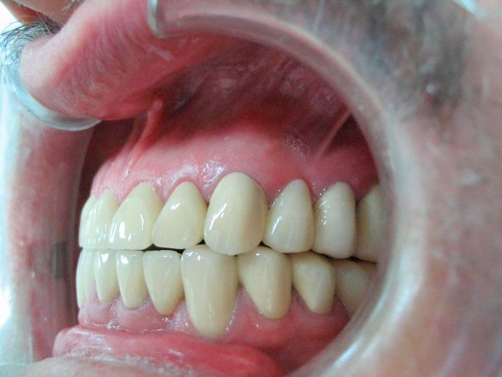
Post-op intraoral left lateral view in occlusion.

**Figure 7.**
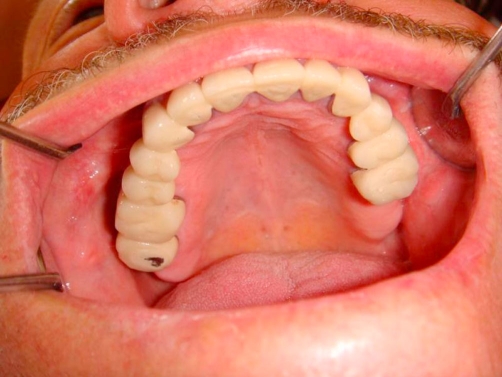
Post-op intraoral occlusal view of upper arch.

**Figure 8.**
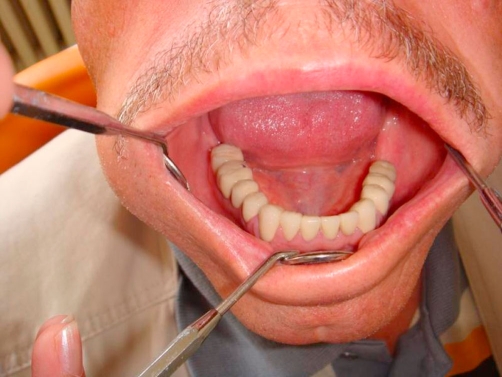
Post-op intraoral occlusal view of lower arch.

**Figure 9.**
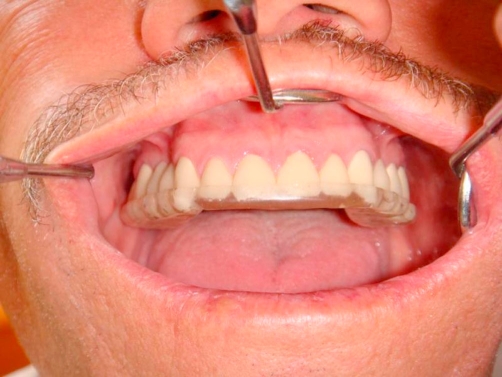
Post-op frontal view with stabilisation splint.

Impressions of both arches were made with irreversible hydrocolloid (Cavex Impressional, Cavex Holland BV: Haarlem, The Netherlands) and poured with a type V dental stone (Glastone 3000, Dentsply International Inc, PA 17405-0872, USA). After determining the occlusal vertical dimension with mounted diagnostic casts and face dimensions, there was decreased vertical dimension. Diagnostic casts were mounted on a semi-adjustable articulator (Stratos 300; Ivoclar Vivadent AG FL-9494 Schaan Liechtenstein, Austria) using a face-bow transfer (UTS3D; Ivoclar Vivadent AG FL-9494 Schaan Liechtenstein, Austria) and a centric relation record (Memosil 2, Heraues Kulzer GmbH Grüner Weg 11-63450 Hanou, Germany). First of all the patient was referred to a gastroenterologist. The epithelial irritation of esophagus was reported in the endoscopic examination and results confirmed GERD. The appropriate pharmacologic agent has been prescribed and the GERD was controlled. After the medical treatment, a maxillary acrylic resin (Vertex Orthoplast, Vertex Dental, Zeist, The Netherlands) splint was fabricated with flat occlusal scheme for the increase of vertical height and for protection of the teeth against further gastric acid and bruxism. Acrylic resin was applied in 1 mm and 2 mm increments to the occlusal surface of the splint over a 30 day period to establish a 3 mm increase in occlusal vertical dimension (OVD). No complaints like muscle pain or discomfort and difficulty in function occurred after the increase in OVD. After re-establishing the vertical dimension, maxillary and mandibular anterior and posterior teeth that were restored with resin-based composite restorations (Filtek Z250; 3M ESPE AG Dental Products, D-82229 Seefeld, Germany) were prepared for metal-ceramic restorations, and laboratory-processed provisional restorations (Luxatemp, DMG 22547 Hamburg, Germany) were fabricated at the new OVD and cemented with zinc-oxide eugenol (Temp-Bond; Kerr Corp). A full contoured wax-up on the mounted casts made it possible to evaluate tooth dimensions to achieve optimal esthetics. Definitive impressions of the prepared maxillary and mandibular teeth were obtained using polysiloxane impression material (Elite HD+, Zhermack 45021, Rouigo, Italy). Working casts were generated from Type IV die stone and mounted into the articulator using interocclusal records. Shade determination (Vita 3D; VITA Zahnfabrik, Bad Sackingen, Germany) was made. Metal-ceramic fixed partial dentures (Wiron 99; Bego,Bremen, Germany/Vitadur Alpha; VITA Zahnfabrik, Bad Sackingen, Germany) were fabricated as full mouth restorations for both maxillary and mandibular arches because of splinting all teeth, evaluated intra-orally, and adjusted (Figures 4, 5, 6, 7 and 8). Because of the diurnal and nocturnal bruxism history of the patient, teeth restored with crowns could be subject to move. The adjacent crowns were splinted to give more support to individual teeth. On the other hand, the patient was clearly informed of the importance of oral hygiene by giving more attention using proximal brushes and dental floss. For lateral movements group disclusion achieved so that masticatory loads would be uniformly distributed on the reconstructed teeth. The full-mouth restorations were cemented with glass-ionomer cement (Meron, Voco, Cuxhaven, 274457, Germany). Also, for bruxism history a laboratory-processed maxillary stabilization splint prescribed for nighttime use was fabricated, adjusted and placed (Vertex Orthoplast, Vertex Dental, Zeist, The Netherland) (Figure 9). The patient was followed up for 2 years without any complaints. The patient’ s esthetic and functional expectations were also satisfied.

## Discussion

Contemporary dentistry offers many treatment options, from conservative to invasive for tooth loss: direct resin composite restorations, laboratory-made adhesive restorations (resin composite, all-ceramic and metal alloys), laboratory-made full crown restorations (metal, metal-ceramics and all ceramics). An acidic environment affects the solubility of dental hard tissue. Gastric contents may have an acidity below pH 1; regurgitation therefore can have a severe demineralizing effect on tooth structure and restorative materials [21]. It was reported that the posterior composite and poly-acid modified composite were effected with respect to surface changes following exposure to simulated gastric juice [22]. Under oral conditions, in the absence of mechanical forces, chemical process or dissolution can produce an increase of surface roughness. These undesirable damages have been associated with the chemical degradation of the surface and subsurface, which may involve either the resin matrix, the filler, or the matrix-filler interface [23]. Due to these facts, metal-ceramic fixed partial dentures was planned for our patient. In his anamnesis, he had paid no attention because of any symptoms affecting his life. But his endoscopic examination showed epithelial irritation.of esophagus and confirmed as silent GERD . Silent GERD is defined as gastric reflux without symptoms such as belching, unexplained sour taste or heartburn [17]. Our patient is with long-standing GERD and may become symptom free but may continue to reflux. Also, due to his neglect for the medical treatment in his history, we assumed him as a self-medicate patient.

## Conclusion

This clinical report describes the dental treatment of a GERD patient by fabricating metal-ceramic fixed partial dentures after his medical diagnosis and treatment.

## Abbreviations

GERD: Gastroesophageal Reflux Disease
OVD: Occlusal Vertical Dimension
